# Molecular Mechanisms of Paraptosis Induction: Implications for a Non-Genetically Modified Tumor Vaccine

**DOI:** 10.1371/journal.pone.0004631

**Published:** 2009-02-27

**Authors:** Neil Hoa, Michael P. Myers, Thomas G. Douglass, Jian Gang Zhang, Christina Delgado, Lara Driggers, Linda L. Callahan, Gerald VanDeusen, Jimmy T. H. Pham, Nirav Bhakta, Lisheng Ge, Martin R. Jadus

**Affiliations:** 1 Diagnostic and Molecular Medicine Healthcare Group, Veterans Affairs Medical Center, Long Beach, California, United States of America; 2 Department of Pathology and Laboratory Medicine, University of California Irvine, Irvine, California, United States of America; 3 Chemistry and Biochemistry Department, California State University Long Beach, Long Beach, California, United States of America; 4 Nursing Department, California State University Long Beach, Long Beach, California, United States of America; 5 Biology Department, California State University Long Beach, Long Beach, California, United States of America; 6 Neuro-Oncology Program, Chao Comprehensive Cancer Center, University of California Irvine, Irvine, California, United States of America; University of Sheffield, United Kingdom

## Abstract

Paraptosis is the programmed cell death pathway that leads to cellular necrosis. Previously, rodent and human monocytes/macrophages killed glioma cells bearing the membrane macrophage colony stimulating factor (mM-CSF) through paraptosis, but the molecular mechanism of this killing process was never identified. We have demonstrated that paraptosis of rat T9 glioma cells can be initiated through a large potassium channel (BK)-dependent process initiated by reactive oxygen species. Macrophage mediated cytotoxicity upon the mM-CSF expressing T9-C2 cells was not prevented by the addition of the caspase inhibitor, zVAD-fmk. By a combination of fluorescent confocal and electron microscopy, flow cytometry, electrophysiology, pharmacology, and genetic knock-down approaches, we demonstrated that these ion channels control cellular swelling and vacuolization of rat T9 glioma cells. Cell lysis is preceded by a depletion of intracellular ATP. Six-hour exposure to BK channel activation caused T9 cells to over express heat shock proteins (Hsp 60, 70, 90 and gp96). This same treatment forced HMGB1 translocation from the nuclear region to the periphery. These last molecules are “danger signals” that can stimulate immune responses. Similar inductions of mitochondrial swelling and increased Hsp70 and 90 expressions by BK channel activation were observed with the non-immunogenic F98 glioma cells. Rats injected with T9 cells which were killed by prolonged BK channel activation developed immunity against the T9 cells, while the injection of x-irradiated apoptotic T9 cells failed to produce the vaccinating effect. These results are the first to show that glioma cellular death induced by prolonged BK channel activation improves tumor immunogenicity; this treatment reproduces the vaccinating effects of mM-CSF transduced cells. Elucidation of strategies as described in this study may prove quite valuable in the development of clinical immunotherapy against cancer.

## Introduction

Dying or dead cells possess distinct, observable and differing morphologies such as autophagy, paraptosis/necrosis and apoptosis [1]. Autophagy consists of cellular self-digestion. Very little is known of the potential vaccinating properties of these dying and dead cells. Paraptosis is thought to be a programmed form of cell death that culminates in cellular necrosis. The molecular mechanisms of paraptosis induction are not well defined. Paraptotic cells are characterized by a process of swelling and vacuolization that begins with physical enlargement of the endoplasmic reticulum (ER) and the mitochondria [2]. The appearance of swollen cells suggests ionic disregulation is followed by water retention. The disruption of intracellular ion homeostasis ultimately causes osmotic lysis. Such lysis releases substances that have been labeled as “danger signals”. These include high mobility group B-1 (HMGB1, also known as amphoretin) [3], heat shock proteins (HSP) [4], and various proteases. Release of these “danger signals” promotes massive inflammation, ultimately stimulating cell-mediated immunity [5]. Lastly, apoptosis is distinguished by nuclear condensation, DNA cleavage, cell shrinkage, membrane blebbing, and HMGB1 retention in the nucleus resulting in apoptotic body formation [6], [7]. Both professional phagocytes and adjacent stromal/parenchymal cells scavenge the apoptotic bodies. Apoptosis has been called the “silent death” because immunological responses are minimized. Antigen presenting cells (APC), after interacting with necrotic tumor cells, produce superior T cell immunizing responses in comparison to apoptotic cells [reviewed in 8]. Dendritic cells (DC) mature more rapidly when exposed to necrotic cells than when exposed to apoptotic cells [9]. Upon exposure to apoptotic cells, interleukin-12 (IL-12) transcription is suppressed in APC [10]. Macrophages and DC fed apoptotic cells produce increased levels of immunosuppressive agents such as prostaglandin E_2_, platelet activating factor, transforming growth factor-β, and interleukin-10 [11]. The presence of these soluble mediators prevented co-stimulatory molecules from being fully expressed by the “activated” APC.

Previously, we reported that rat T9 and human U251 glioma cells, along with mouse Hepa1-6 hepatoma cells retrovirally transduced with a unique, spliced variant of the membrane form of the macrophage colony stimulating factor (mM-CSF) were killed *in vitro* by monocytes/macrophages [12]–[17]. *In vivo* studies confirmed the lack of tumorigenicity of these mM-CSF transduced tumor cells in animals, even when 5–10 million tumor cells were injected [18]–[20]. After 4 hours the mM-CSF transduced cells could be morphologically identified as paraptotic cells; after 12–18 hours increased HSP expression was observed [14], [15]. It was hypothesized that this killing process allows tumor immunity to be stimulated via the release of various molecules that serve as “danger signals”. This peripheral immunization resulted in systemic immunity that induced tumor rejection in either subcutaneous or established intracranial tumors [18], [19].

In this study, a molecular mechanism is presented for the ability of rat monocytes to kill mM-CSF expressing T9 glioma cells by producing a disruption of ionic homeostasis in the targeted cell. Data from the present study demonstrates that osmotic disregulation of the tumor cells induced by big potassium (BK) channel activation, provides not only the mechanism by which macrophage-mediated paraptosis occurs but also how immunity using paraptotic cells is subsequently initiated.

## Results

### mM-CSF T9 tumor cells die of a swelling/vacuolization process called paraptosis and reactive oxygen species derived from macrophages can kill T9 cells

**Figure 1:**
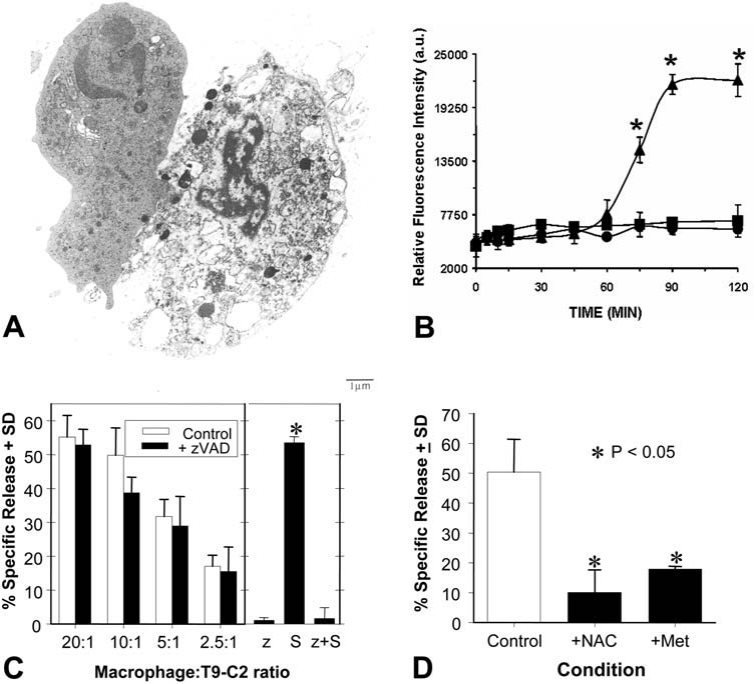
T9-C2 cells die *in vivo* via paraptosis and reactive oxygen species (ROS) derived from macrophages kill T9 cells *in vitro*. Panel A: Five million T9-C2 cells were injected subcutaneously into a syngeneic rat 4 hours earlier. Magnification 3,000×. Panel B shows the kinetics of ROS induction within cultures of rat peritoneal macrophages responding to mM-CSF expressing T9 cells (T9-C2) at Time 0. The time kinetics of the chemiluminescence produced by H_2_DCFDA pre-labeled macrophages in response to the T9-VV (squares) or T9-C2 (triangles) or the macrophages alone (circles) are demonstrated. Panel C: Macrophage-mediated cytotoxicity against the mM-CSF expressing T9-C2 cells is not prevented by a caspase inhibitor, zVAD-fmk. Rat peritoneal macrophages were added at various macrophage∶tumor ratios (20∶1, 10∶1, 5∶1, 2.5∶1) with H^3^ labeled T9-C2 cells in a 24 hour release assay. Ten µM zVAD-fmk was added at the start of the incubation. Ten micromolar zVAD-fmk inhibited the cytotoxicity of staurosporine induced apoptosis of T9-C2 cells. The right subpanel shows the cytotoxicity of the glioma cells in the presence of 10 µM zVAD-fmk (z), staurosporine (S) or the combination of both zVAD and staurosporine (z+S). Panel D: Twenty mM N-actetyl-cysteine (NAC) and 1 mM methimazole were added to the cultures (at a 10 macrophage: 1 T9-C2 cell ratio) at time 0. The asterisks denote significant differences between experimental and control values (P<0.05).

When the rat T9 glioma cells expressing mM-CSF (T9-C2 cloned cells) were injected subcutaneously, all the transduced T9 glioma cells died by a paraptotic process resulting in osmotic lysis (Figure 1A). Paraptotic morphologies were not seen to develop when the unmodified T9 cells were injected. The untreated T9 cells formed subcutaneous tumors. Throughout our work over the past 10 years, no T9-C2 cells have escaped this type of destruction, a cytolytic process mediated by the myeloid cells *in vivo*, by forming a tumor. More importantly, once the animals eliminated these paraptotic tumors at a subcutaneous site, tumor specific immunity resulted either at subcutaneous or intracranial sites [16], [18], [19].

From three separate experiments, a representative illustration emerges for the time kinetics of a rat peritoneal-derived macrophage (pre-labeled with H_2_DCFDA) response to the T9 and T9-C2 cells. The response was monitored using a luminometer to measure the production of reactive oxygen species (ROS) (Figure 1B). The singly cultured macrophages or the macrophages cultured with the T9-Viral Vector (T9-VV) cells demonstrated flat baseline values. At 75 minutes, a significant (P<0.05) elevation of the ROS generated by the macrophages responding solely to the mM-CSF expressing T9 cells began. This strong degree of ROS induction continued over the next 120 minutes until the experiment was concluded.

To demonstrate that this cytotoxicity was not due to an apoptotic-dependent pathway, cytotoxicity experiments were performed in the presence of the broad-caspase inhibitor, zVAD-fmk. In the presence of 10 µM zVAD-fmk, the cytotoxocity of the macrophages against the T9-C2 cells was not inhibited (Figure 1C), indicating that the killing process is not mediated via a caspase- dependent pathway. Increasing the amount of zVAD-fmk up to 50 µM did not change the results (data not shown). As a control, zVAD-fmk was found to completely inhibit staurosporine induced apoptosis of the T9 glioma cells (Figure 1C, right panel).

Cytotoxicity studies were performed using H_2_O_2_ and HOCl, which activate H_2_DCFDA fluorescence, in order to prove that these macrophage derived products directly killed T9 glioma cells. T9 cells were effectively killed within 16 hours by the H_2_O_2_ and hypochlorite ions (0.25 mM) produced by the macrophages in the respiratory burst (data not shown). The ROS scavengers, N-acetyl cysteine and methimazole, prevented the cytotoxic effects of the macrophage derived ROS on the macrophage mediated cytotoxicity of the mM-CSF (Figure 1D). Both of these scavengers significantly inhibited the macrophages' ability to kill the mM-CSF expressing T9 cells. In summary, these data from these experiments show that ROS are mediators of this cytotoxicity, but do not totally explain the molecular mechanism of paraptosis induction.

### Functional BK channels are found on the membrane, mitochondria and ER of the T9 cell

Many glioma cells express BK channels [21]–[23]. Oxygen has been proposed to regulate BK channel activity [24]. In the presence of O_2_, or other oxygen species, an electron is transferred from heme by NADPH 450 reductase to hemoxygenase-2, thereby enzymatically generating carbon monoxide (CO). The presence of CO activates the BK ion channels permitting the efflux of K^+^ ions from the intracellular pools. Since the monocytes, in response to membrane M-CSF found on T9 glioma cells, produce a respiratory burst containing various oxygen species, it was hypothesized that this is the initial event inducing cell death by activation of BK channels.

**Figure 2:**
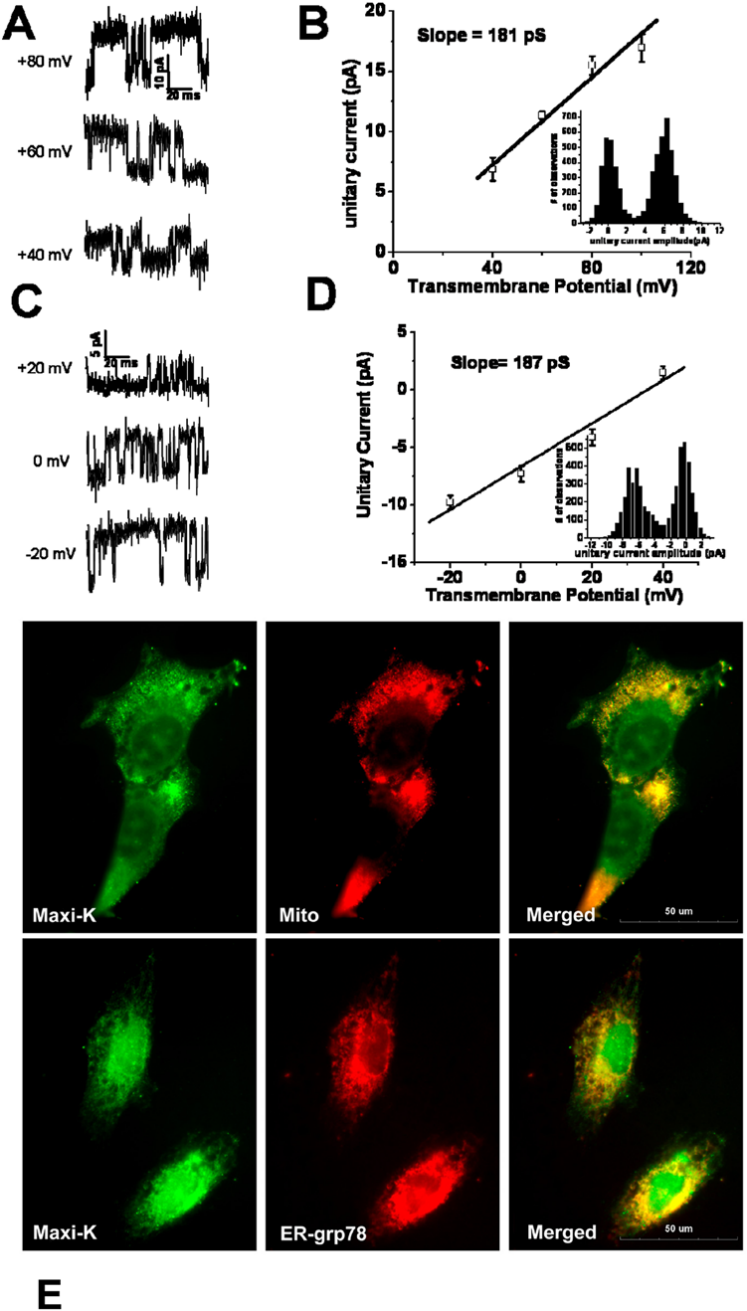
T9 glioma cells possess BK channels on the cell membrane and within the mitochondria and ER. T9 glioma cells possess functional BK channels as demonstrated by patch-clamping techniques. Cell attached recordings of BK channels in T9 cells. Panel A: representative traces from a cell-attached patch in response to depolarizing pulses. Panel B: unitary current-voltage relationship of the channels at positive potentials. Inset is an amplitude histogram (bin width is 0.5 pA) obtained at a transmembrane potential of +40 mV. The slope of this line indicates a conductance of 181 pS. Panel C: representative traces from a cell-attached patch in response to hyperpolarizing pulses. Panel D: unitary current-voltage relationship of the channels at mainly negative potentials. Error bars represent the mean values +/− the SEM for 3 separately patched cells (n = 3). Inset is an amplitude histogram (bin width is 0.5 pA) obtained at a transmembrane potential of 0 mV. The slope of this line indicates a conductance of 187 pS. Panel E: Top row: Left panel demonstrates cells viewed with exposure to only the anti-BK channel antibody; Middle Panel: The cells viewed have been treated with Mito-tracker Red; Right Panel: The merger of the cells in the left and middle panels. Bottom row: Left Panel demonstrates another preparation of the T9 glioma cells stained with BK channel antibody. Middle Panel contains cells stained with anti-GRP78 antibody (to identify the ER) and PE-conjugated secondary antibody, indicative of the ER. Right Panel: The merged yellow figures represent one plane where co-localization has occurred.

Standard patch clamping techniques demonstrated that T9 glioma cells have functional cell-surface BK channels. Cell attached recordings of the T9 glioma cells (Figure 2, Panels A–D) revealed large conductance channels consistent with BK channels as previously characterized within glioma cells in other studies [21]–[23]. The presence of BK channels was demonstrated using both depolarizing (Panel A) and hyperpolarizing pulses (Panel C). The resulting conductance currents demonstrated strong voltage dependence and single-channel currents were resolved in these patches. The unitary slope conductance of the single-channel currents was 181 pSiemens (pS) when a positive potential (Panel B) was applied, while the conductance was 187 pS when negative potentials (Panel D) were used. The transmembrane potential in the cell-attached patch configuration was measured because the membrane was intact and the actual membrane potential was unknown. The membrane potential was estimated by breaking the patch at the end of the experiment and the average value from whole-cell recordings was used. These results indicated that T9 glioma cells express functional BK channels at the cell surface.

Co-localization studies identified the intracellular locations of the BK channels. The results are presented in Figure 2. The cells were pre-stained with a Mito-tracker red (Top Row of the fluorescent micrographs, Middle Panel) to specifically identify the mitochondria, while the BK channels were identified using the green (FITC) fluorescent anti-BK channel antibody (Top Row, Left Panel). The merged figure (Top Row, Right Panel) shows yellow staining in many sites within the cells where the mitochondria and BK channels are found to be co-localized. Additional sites of green fluorescence were interpreted as marking non-mitochondrial organelles such as the endoplasmic reticulum (ER). The ER was identified with red fluorescent mouse anti-GRP78 antibody (Lower Row, Middle Panel). BK channels and the ER also co-localized together (Bottom Row, Right Panel). Thus, BK channels are found within these 2 organelles as well. This substantiates the presence of BK ion channels in multiple sites within the T9 glioma cells.

### BK channel activators induce formation of vacuoles derived from the mitochondria and endoplasmic reticulum in the T9 glioma cells

It was hypothesized that a forced opening of the BK channels within the T9 glioma cells as a result of ROS from the monocytes, disrupts the normal internal ion homeostasis of the targeted cell. Once activated, BK channels allow an osmotic imbalance to develop, thereby initiating the vacuolization process seen in paraptosis. The BK channel activators, phloretin or pimaric acid, were used to determine if cell vacuolization could be induced after 1 hour of treatment. Adherent T9 glioma cells were exposed to BK channel activators. After 1 hour of phloretin treatment these cells became vacuolated (Figure S1, Panel B). Similar results were observed with the use of pimaric acid (Figure S1, Panel C). Non-treated T9 glioma control cells remained healthy, adherent cells (Figure S2, Panel A).

T9 cells were also stained with either Mito-Tracker (Figure S1, Panels D, E and F) or ER- Tracker (Panels G, H and I) to specifically stain the mitochondria or the endoplasmic reticulum, respectively. The untreated T9 cells displayed small delicate red mitochondria throughout the cell (Panel D). In contrast, within the phloretin treated T9 cells (Panel E) or pimaric acid treated T9 cells (Panel F) enlarged red mitochondria are seen. Similar swelling effects in the ER were seen when the T9 cells were pre-labeled with ER-Tracker and then treated with either phloretin (Panel H) or pimaric acid (Panel I). The amount of the area of either green or red fluorescence of the organelles was quantitated at the pixel level using the Compix software to measure the size of the fluorescent area. These data are presented in Panel J. Both phloretin and pimaric acid caused significant enlargement of these organelles. Thus, it appears that BK channels are functional within these organelles.

### Ultrastructural analysis confirms that the mitochondria and the ER are targeted by sustained BK channel activation

**Figure 3:**
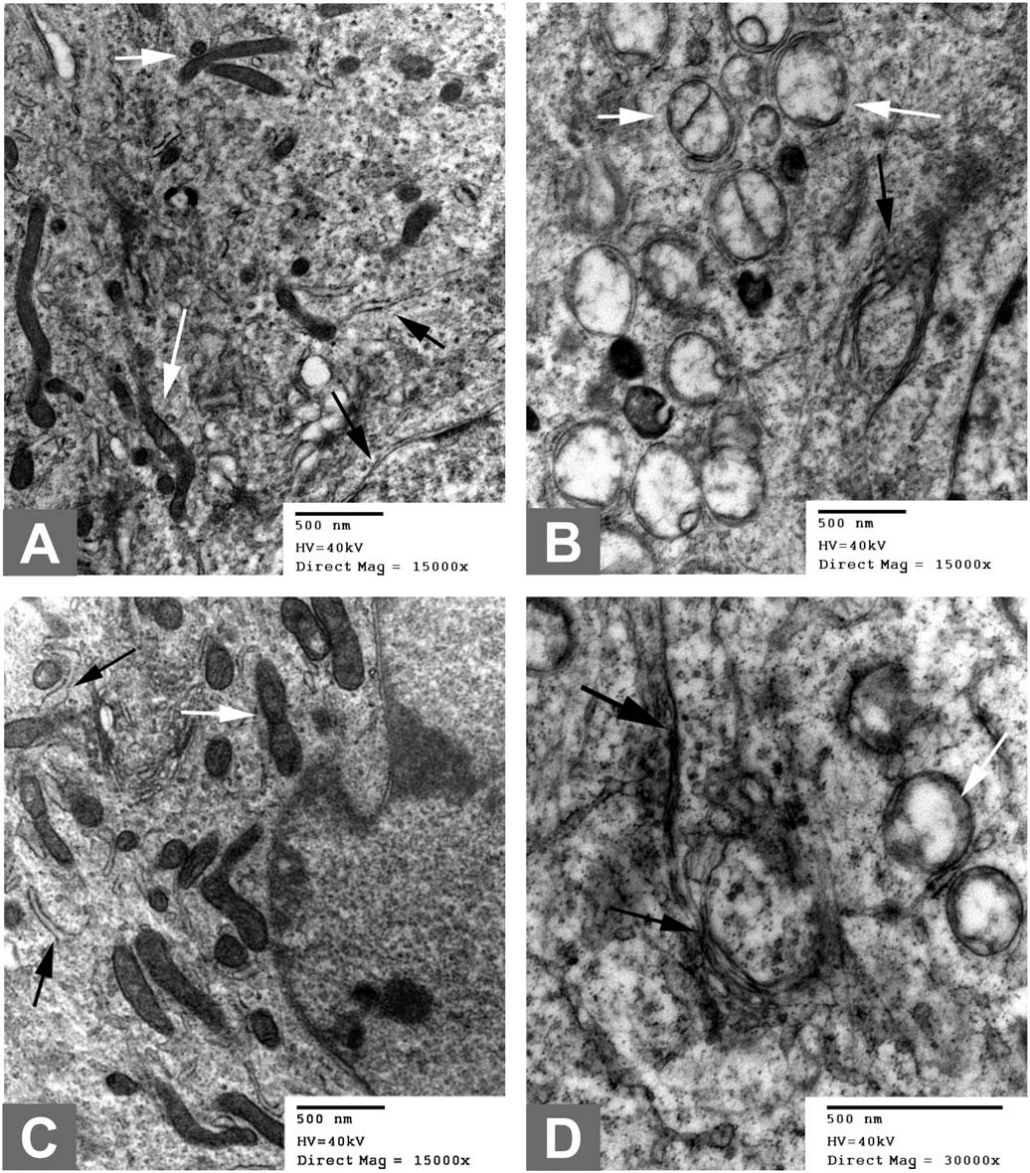
Electron microscopy reveals phloretin and pimaric acid cause the swelling of the mitochondria and the endoplasmic reticulum. T9 cells were incubated without any BK channel activators (Panel A); 1 hour with 0.01 mM pimaric acid (Panel C); 1 hour with 1 mM phloretin (Panels B and D). All magnifications are 15,000×, except Panel D which was 30,000×. The white arrows indicate the mitochondria, while the black arrows show the endoplasmic reticulum.

Electron microscopic studies were done to confirm that the mitochondria and the endoplasmic reticulum were indeed affected by prolonged BK channel activation. Figure 3 shows the ultrastructures of T9 cells treated for 1 hour with no BK channel activation (Panel A); as well as T9 cells treated with 0.01 mM Pimaric acid (Panel C); and those treated with 1 mM phloretin (Panels B and D). Non-BK channel activated T9 cells display normal mitochondria (white arrows) and normal ER (black arrows). T9 cells treated with the pimaric acid do show that both organelles are swollen, with the cristae of the mitochondria becoming diffuse. The cells treated by phloretin appear to be much more advanced in the development of pathology with vacuoles clearly forming that have pushed the cristae up against the inside of the swollen mitochondria. This finding suggests that the contents of the inner mitochondria have been physically been changed. This alteration then exerts some pressure that pushes out the cristae. All the swollen organelles contain electron dense material, a finding that eliminates any possibility that macropinocytic bodies were the source of these vacuoles.

### BK channel activators and carbon monoxide are observed to induce T9 cells swelling; r-iberiotoxin prevents the actions of BK channel activators on these cells

**Figure 4:**
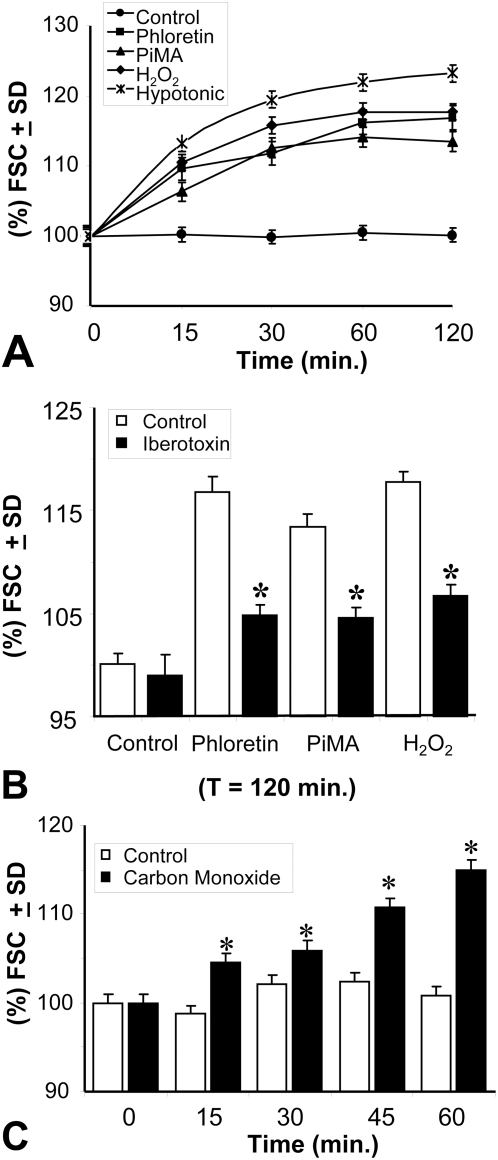
BK channel activators and carbon monoxide induce T9 cells to swell. T9 cells in suspension were incubated with 1 mM phloretin, 0.01 mM pimaric acid, 1 mM H_2_O_2_ or in a hypotonic PBS (0.9× PBS) at 37°C. At timed intervals, the samples were analyzed on the flow cytometer. All data from the experimental groups are significantly different (P<0.05) from the untreated cells (black circles). Panel A shows the time kinetics; Panel B analyzes the data at 120 minutes and demonstrates the effect of 0.05 µM r-iberiotoxin in preventing cell swelling. Panel C shows that T9 cells swell in response to CO saturated media. All the experimental treatments in Panel A were significantly different (P<0.05) from the control cells. The asterisks in Panels B and C indicate a significant difference (P<0.05) between the treated cells and their respective controls.

Flow cytometry using a forward scatter (FSC) parameter (indication of size) demonstrated that T9 cells swelled in response to the BK channel activators. In response to phloretin, and pimaric acid, the T9 cells quickly enlarged 6–10% within 15 minutes, and continued to enlarge by as much as 112–118% over the next 105 minutes (Figure 4A). Size increases were also observed when H_2_O_2_ and a hypotonic solution (0.9× PBS) were added to the cells. During the course of this experiment, additional experiments were performed in which r-iberiotoxin was added to the cultures. At the 120 minute mark, representative data are shown in which iberiotoxin (when added at Time 0) significantly inhibited cellular swelling (Figure 4B) to both BK channel activators: phloretin and pimaric acid. R-iberiotoxin also significantly prevented H_2_O_2_-treated T9 cells from swelling.

It has been reported that CO acts as a secondary messenger that directly opens the BK channel. Experiments were performed in which T9 cells were exposed to CO saturated media. Over a period of exposure time, varying from 15 to 60 minutes, T9 cells progressively swelled upon prolonged exposure to the CO saturated media (Figure 4C). This data supports the hypothesis that CO can act as a secondary messenger and directly activates the BK channels.

### Replacing Na^+^ ions with K^+^ ions prevents the T9 cells from swelling in response to phloretin

**Figure 5:**
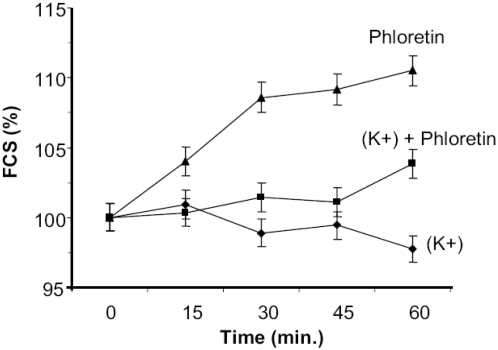
T9 cells fail to swell in response to BK channel activation when Na^+^ ions are replaced by K^+^ ions. T9 cells were cultured in regular PBS (high Na^+^ ions and low K^+^ ions) and stimulated with 1 mM phloretin (triangles). T9 cells were incubated in PBS where the Na^+^ were replaced with K^+^ ions and then cultured alone (diamonds) or stimulated with 1 mM phloretin (squares) at 37°C. At the times indicated, samples were analyzed by flow cytometry for size using the forward scatter parameter.

When BK channels are opened, intracellular K^+^ ions are released, permitting Na^+^ ions with water to enter the cell or organelles to maintain electroneutrality. This infused water ultimately causes the cells and organelles to swell. Experiments were conducted using PBS, in which the Na^+^ ions were replaced with an equivalent amount of K^+^ ions to maintain osmolarity. Upon BK channel activation, the K^+^ ions will escape, while little or no Na^+^ ions and water could enter the cell. Figure 5 shows that when using standard PBS (high Na^+^ and low K^+^), T9 cells swelled in response to phloretin. When K^+^ enriched PBS (low Na^+^ and high K^+^) was substituted, the T9 cells maintained normal size for 45 minutes, with some shrinkage to 97.5% after 60 minutes. These values were not statistically, significantly different from those obtained at the previous time points. When the T9 cells were exposed to phloretin in the K^+^ enriched PBS, very little cell swelling occurred. These values were significantly different (P<0.05) from those T9 cells stimulated with the phloretin while in the normal PBS. Hence, it appears that the influx of Na^+^ ions is largely responsible for T9 cell swelling in response to phloretin.

### Cell cytotoxicity required 17 hours of continuous BK channel activation and was prevented by BK gene knock-down

**Figure 6:**
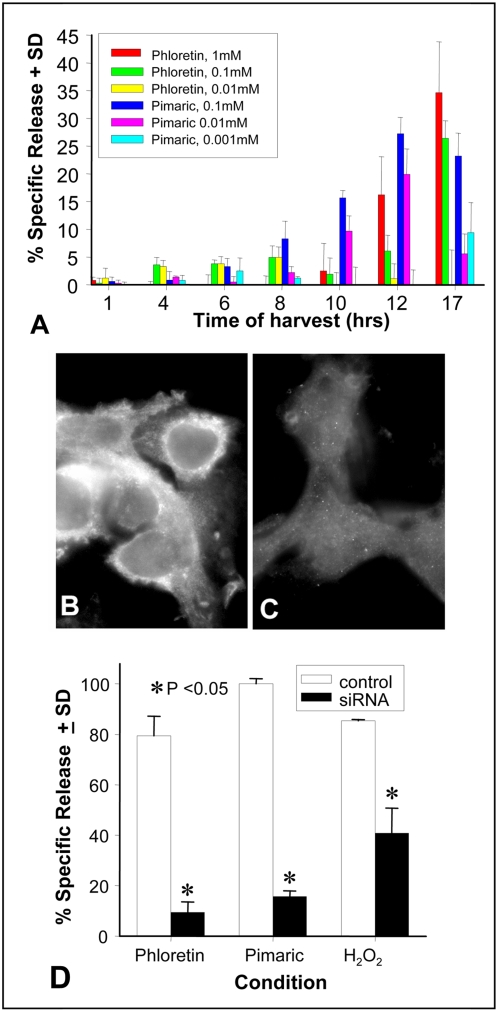
Phloretin and pimaric acid kill the T9 cells and can be prevented by genetic knock-down. Panel A: One million Cr^51^ labeled T9 glioma cells were incubated with 1.0, 0.1 or 0.01 mM phloretin or 0.1, 0.01 or 0.001 mM pimaric acid in 24 well plates. Data shown at the indicated times show the percent of specific release, ±standard deviation of triplicate cultures. T9 cells were transfected with either 80 pmol BKα or siRNA control. Two days later aliquots of the cells were immunologically stained with the anti-BK channel antibody (Panel B: siRNA control; Panel C: BK siRNA treated). The data in Panel D demonstrates the cytotoxicity of the T9 cells in response to 1 mM phloretin, 0.01 mM pimaric acid, or 1 mM H_2_O_2_ for 24 hours. The asterisks indicate significant differences (P<0.05) between the experimental and their respective controls.

The time at which cytotoxicity occurred was determined by performing Cr^51^ release studies (Figure 6, Panel A). No cytotoxicity occurred after 4 hours of exposure, even though the cells gave the appearances that they were greatly stressed after 1 hour of treatment. Even so these data indicated that the cells' membranes were intact and retained the radioisotope. At 8 hours, cytotoxicity was beginning to be detected, although the amount of cellular death was not considered significant. By 12 hours, obvious signs of cytotoxicity were noted and were observed to be maximal at 17 hours in cells exposed to the two highest concentrations of phloretin (1.0 mM and 0.1 mM) and pimaric acid (0.1 mM). Thus, actual cell death required an extended time of exposure in addition to the initiation time for swelling and vacuolization.

Genetic methods confirmed that the BK channels were responsible for the observed cytotoxicity. Invitrogen's Stealth siRNA construct was used to specifically knock-down the rat BKα channels. Two days after siRNA transfection, quantitative real-time PCR indicated that the amount of mRNA for BK channels was reduced by 86% when compared to a non-specific siRNA control (0% loss). The loss of BK channel protein expression was confirmed by immunofluorescence (Figures 6B and 6C). When cytotoxicity studies were carried out for 24 hours (Figure 6D), phloretin, pimaric acid and H_2_O_2_ killed the siRNA control T9 cells; whereas, the BKα siRNA knocked-down T9 cells were resistant to the cytolytic effects of the BK channel activators. Hence the transient knockdown of the BK channels made the T9 cells resistant to the various cytotoxins.

### Loss of intracellular ATP coincides with the time of the T9 cells death

**Figure 7:**
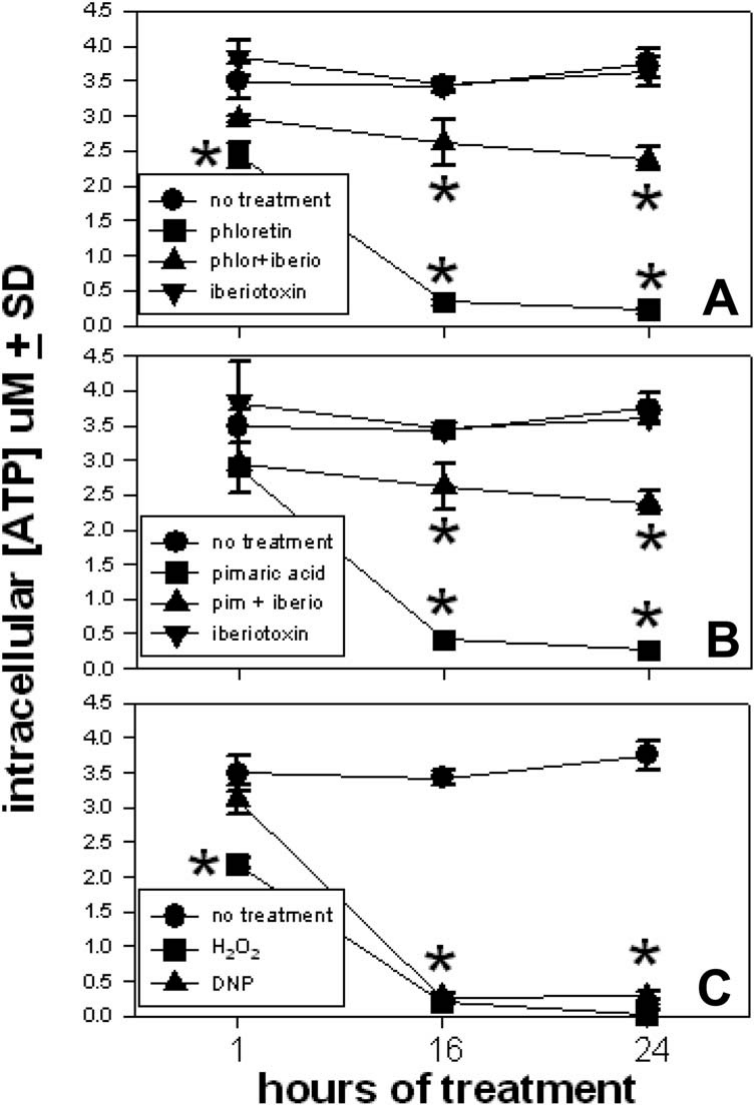
BK channel activators cause a loss of intracellular ATP with the T9 cells. T9 cells were cultured in either 1 mM phloretin, 0.01 mM pimaric acid, 0.05 µM r-iberiotoxin, 1 mM H_2_O_2_ or 0.1 mM DNP in 96 well plates. After 1, 16 or 24 hours the cells were lysed and the amount of ATP was measured using a luciferase/luciferin-based assay. The asterisks indicate a significant difference (P<0.05) of the experimental values from those results of either the untreated or iberiotoxin treated cells.

For cells to maintain proper cation homeostasis, a Na^+^/H^+^ antiporter or a Na^+^/K^+^ exchanger is used to actively pump out Na^+^ ions. Both Na^+^ removal transporters are ATP-dependent. The mitochondria are the source of the required ATP production. By targeting the mitochondria through BK disregulation, the resultant reduced efficiency of ATP production may be seen as a mechanism whereby cell lysis proceeds. The levels of intracellular ATP were measured at various times to determine if ATP was being lost as a result of prolonged BK activation. Figure 7A illustrates that the levels of ATP remained constant for 24 hours when T9 cells were cultured alone or if cultured with iberiotoxin. One hour after phoretin addition a significant loss of intracellular ATP levels was noted. When iberiotoxin was added, the loss of ATP was almost completely mitigated, but ATP levels were not restored to control levels. Identical results were obtained when pimaric acid was substituted for phloretin (Figure 7B). H_2_O_2_-treatment also produced the immediate loss of ATP (Panel C). As a specific control, 2,4 dinitrophenol (DNP) was used as a mitochondrial respiration inhibitor. By visual observation it is noted that H_2_O_2_-treated cells swelled, while the DNP-treated cells did not swell. This indicates that simple mitochondrial inhibition is not sufficient to induce cellular swelling. Additionally, BK channel activation is also needed in addition to ATP depletion, to fully display the same cellular pathology.

### Mitochondrial enlargement occurs when the macrophages specifically contact the T9-C2 cells

Studies were conducted in which the T9 or T9-C2 cells were pre-labeled with MitoTracker red and then exposed *in vitro* to macrophages (not pre-labeled for identification purposes) for 4 hours. Figure S2 demonstrates that macrophages labeled with a green nuclear dye show contact with either T9 cells (Panel A) or T9-C2 cells (Panel B). In Panel A, two macrophages are in contact with a T9 cell and the mitochondria retain their normal size. In contrast, the T9-C2 cell displays enlarged red mitochondria when two macrophages interact with it. This would indicate that mitochondria of the mM-CSF target cells are specifically affected by the activated macrophages.

### Iberiotoxin prevented macrophage-mediated death of mM-CSF T9-C2 cells

It was demonstrated that macrophage mediated killing of mM-CSF expressing T9-C2 glioma cells could be prevented by BK channel inhibition of the target cells. The macrophages, at a 10∶1 macrophage∶tumor ratio, produced a 66.8±3.8% specific release of T9-C2 cells, while the presence of the iberiotoxin reduced the incidence of cellular death to 16±4.9% (Figure S3). The presence of iberiotoxin significantly (P<0.005) reduced lysis of the T9-C2 cells by 76%, verifying that macrophage-mediated cytotoxicity of the mM-CSF expressing glioma cells manifests itself largely through a BK channel-dependent pathway.

### Macrophages contacting the T9-C2 cells produced a lowered intracellular ATP level

Our model of the process by which macrophages kill the mM-CSF expressing T9 cells postulates that the depletion of intracellular ATP eventually leads to cell lysis. This occurs because of the prevention of the sodium ion efflux, either by the actions of a sodium anti-porter or a sodium/potassium ATPase exchanger, which requires ATP for transporter function. When intracellular ATP levels are sufficiently depleted, the cell's membrane should osmotically rupture due to lack of the ability to pump out the unwanted water and Na^+^ ions. This proposed model then requires a prolonged time period for cytotoxicity to occur. Hence, it is necessary to demonstrate that the intracellular ATP levels are depleted prior to cellular death.

**Figure 8:**
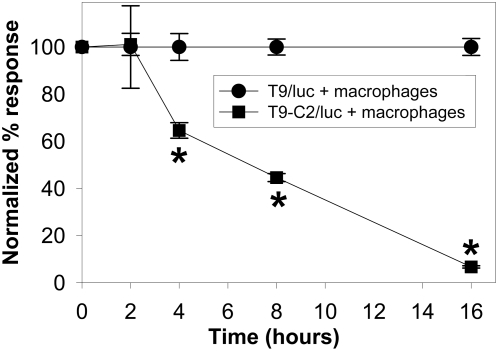
Macrophages cause a loss of intracellular ATP with the T9-C2 cells. T9/luc or T9-C2/luc cells were cultured with an equal number of macrophages in a 96 well plate, after 2, 4, 8 or 16 hours the cultures were pulsed with luciferin and the amount of ATP was measured using a luminometer within 15 seconds. Since the baseline values (time 0) of the T9/luc cells had a higher value than the T9-C2/luc cells, the data was normalized to reflect 100% of baseline values. The asterisks indicate a significant difference (P<0.05) of the luminescence of the T9-C2 cells responding to the macrophages from those results of the macrophages responding to the T9/luc cells.

The only way to conclusively prove this scenario is correct, was to use a retroviral luciferase-based assay. Luciferase produces luminescence when the cell-permeable substrate, luciferin, is added and ATP is present within the cell. The T9-C2 and T9 cells were also transduced with the luciferase gene. Experiments were done in which the T9/luc+ and T9-C2/luc+ clones were incubated with the macrophages for various lengths of time to determine when the intracellular ATP levels were depleted. The kinetics of ATP depletion are shown in Figure 8. Starting at 4 hours there was a significant reduction in the intracellular ATP levels of the T9-C2/luc cells reacting to the macrophages when compared to those T9/luc cells responding to the macrophages. By 8 hours there was only 44% of the initial amount of the ATP levels within the T9-C2/luc+ cells. And by 16 hours, only 4% of the luminescence was present in the T9-C2/luc cells reacting to the macrophages. The loss of intracellular ATP that was observed before cell death occurred at 16 hours may be seen in Figure 6A.

### Prolonged BK channel activation induces the release of “Danger Signals”

Previously, when mM-CSF transduced glioma cells were injected into rodents, tumor cell growth was not observed. Tumor cells, closely examined within 1 day after injection, were noted to have died of paraptosis (Figure 1A). Immunohistology also revealed an increased expression of many “danger signals” such as heat shock proteins and nitrotyrosines (indicative of peroxynitrite) [14], [15], that can stimulate inflammation and immunity. It can be speculated that this process of tumor cell death sets up the proper conditions in which the immune system can fully stimulate anti-tumor immune responses [14], [15], [18], [24]. If a paraptotic mechanism of death for mM-CSF transduced tumor cells is correct, then paraptotic T9 cells (without mM-CSF expression) killed by prolonged BK channel activation should reproduce tumor immunity.

**Figure 9:**
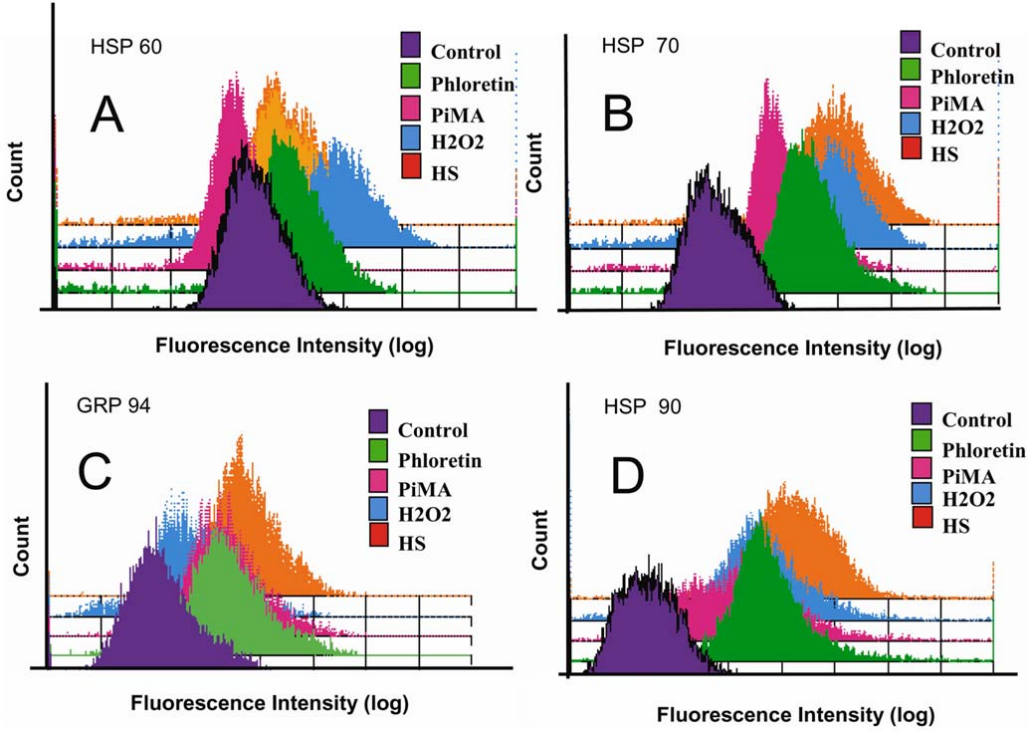
BK Channel activators stimulate a HSP response. T9 cells were incubated 1 mM phloretin, 0.01 mM pimaric acid or 1 mM H_2_O_2_. As a positive control, T9 cells were heat shocked 43°C for 5 minutes and returned to 37 C. After 6 hours, the T9 cells were fixed, permeabilized and stained for heat shock proteins Hsp60 (Panel A), Hsp70 (Panel B), Grp94 (Panel C) and Hsp90 (Panel D).

T9 cells were exposed to prolonged BK channel activation for a period of 6 hours and then tested by intracellular flow cytometry for the presence of “danger signals” like the heat shock proteins (Hsp60, 70, 90 and GRP94/gp96). T9 cells were incubated in the presence of the BK channel activators: phloretin or pimaric acid. In addition, H_2_O_2_ was used as well to simulate macrophage ROS responses. To demonstrate maximal heat shock responses, a set of T9 cells that were heat-shocked for 43°C for 5 minutes were added as a positive control. Representative data from repeated experiments are presented in Figure 9.

For all the T9 cell populations treated with either phoretin, pimaric acid, or H_2_O_2_, the expression of intracellular Hsp60 (Panel A), Hsp70 (Panel B), Grp94 (Panel C) and Hsp90 (Panel D) was elevated. Most of these BK channel-activated responses were almost equivalent to those responses produced when the T9 cells were heat-shocked for 5 minutes. The only exception was the response of T9 cells reacting to pimaric acid for Hsp60 expression.

**Figure 10:**
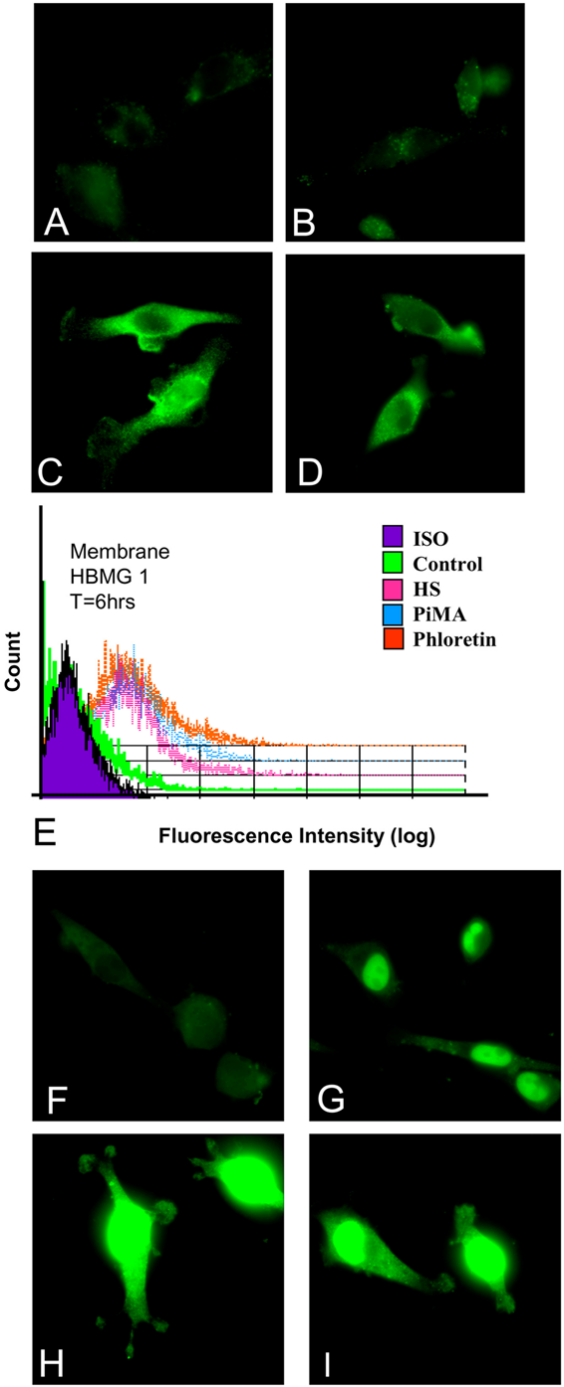
HMGB1 translocates from the nucleus to the membrane as a result of BK channel activation or by hydrogen peroxide. Panels A–D displays the expression of HMGB1 of non-permeablized T9 cells. T9 cells were treated under the various conditions for 6 hours. Panel A shows untreated T9 glioma cells stained with only the secondary antibody. Panel B are control non-treated T9 cells stained for anti-HMGB1 antibody (green). Panels C and D shows T9 cells treated for 6 hours with either 1 mM phloretin, or with 0.01 mM pimaric acid, respectively. To confirm that HMGB1 has been translocated to an extracellular location, flow cytometry confirmed that the HMGB1 (Panel E) was on the extracellular surfaces. Another set of T9 cells were treated under identical conditions as described above (Panels A–D). Panels F–I displays the same treatment of cells shown in Panels A–E, except these cells were first permeabilized before the staining was done. In all cases the HMGB1 has translocated from the nucleus/perinuclear regions through the cytoplasm towards the edge of the membranes of the effected cells.

Another possible “danger signal” that can be detected in T9 cells treated by the BK channel activation is the release of HMGB1 from the perinuclear location with subsequent translocation to the cells' peripheral membranes (Figure 10). This translocation was seen not only with the permeabilized cells (Figure 10, Panels H and I), but also in non-permeabilized cells (Panels C and D). Cell surface expression of HMGB1 was also confirmed by doing flow cytometry on non-permeabilized cells (Panel E).

### BK channel activation produces similar results with the F98 glioma cell

**Figure 11:**
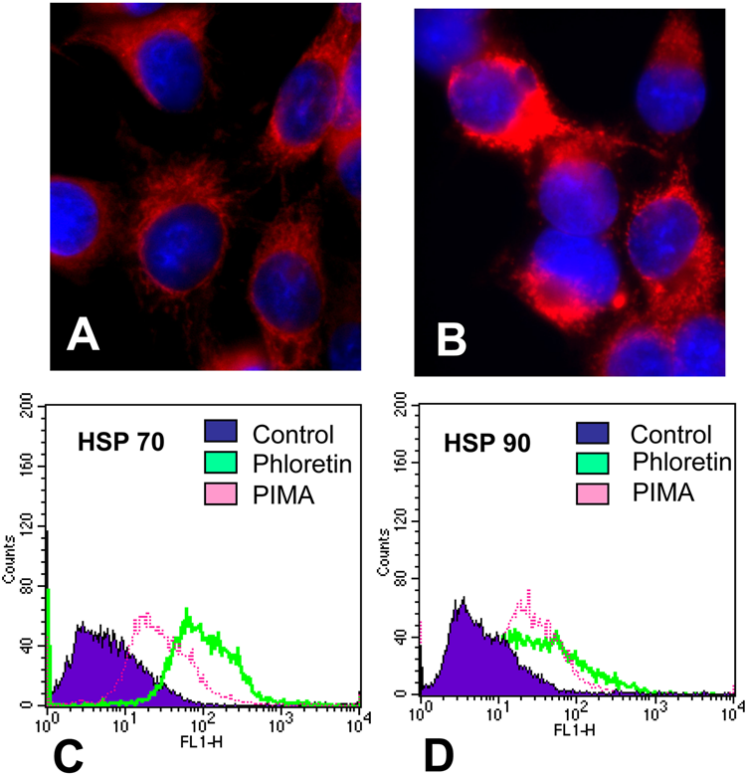
Phloretin induces mitochondrial swelling and Hsp70 and 90 expression within F98 gliomas. Mitotracker labeled F98 glioma cells were exposed to culture media alone (Panel A) or exposed to 1 mM phloretin for 30 minutes (Panel B). F98 cells were exposed to 1 mM phloretin or 0.1 mM pimaric acid for 6 hours and then stained for the presence of Hsp70 (Panel C) or Hsp90 (Panel D) by using intracellular flow cytometry.

Data was generated that demonstrated that induction of mitochondrial swelling along with enhanced Hsp70 and 90 expression could be reproduced in another rat glioma cell line, F98. In response to phloretin, F98 cells swelled within 15–20 minutes (data not shown), while concurrently the mitochondria swelled within 30 minute to exposure with phloretin (Figure 11B). When F98 cells were exposed to either phloretin or pimaric acid for 6 hours, more Hsp 70 and 90 were expressed (Figure 11C+D). These results indicate that BK channel activation induced-pathology is reproducible using a different rat glioma cell line.

### Dendritic cells mature in response to BK channel activated/killed T9 cells

**Figure 12:**
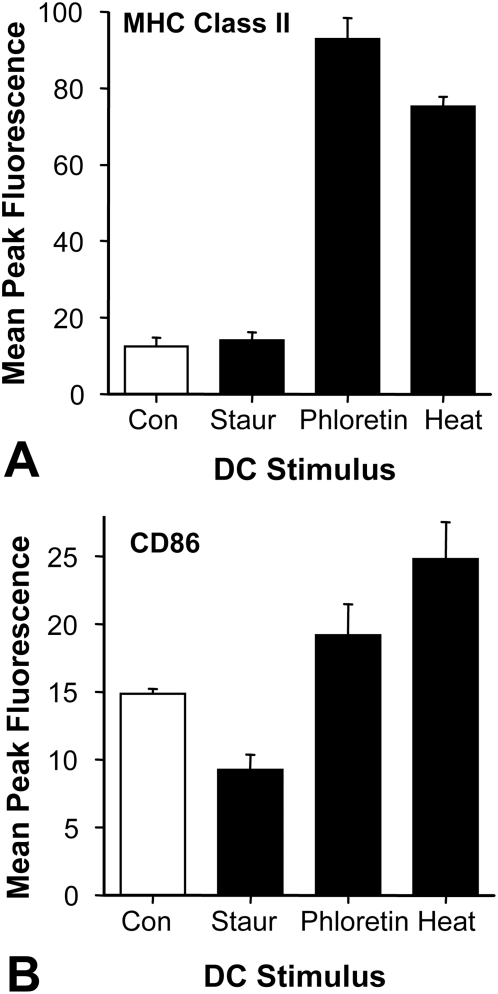
BK channel activated/killed T9 cells induce the maturation of the immature DC. Three day old rat bone marrow dendritic cells were exposed for 1 day to T9 cells that were killed by exposure to: 10 µM staurosporine killed T9 cells (Staur), 1 mM phloretin (Phlor), or heat shocking for 5 minutes followed by 18 hour exposure to 0.9× PBS (Heat). The DC were stained for cell-surface dendritic cell maturation surface markers: MHC class II antibodies or CD86. Ten thousand cells were analyzed on the flow cytometer and compared to the untreated control cells (Con). The data from the mean channel peak number±standard deviation is compared.

BK channel activated/killed T9 cells were next used to stimulate the maturation of immature bone marrow derived dendritic cells *in vitro*. Immature rat bone marrow dendritic cells were grown for 3 days in recombinant interleukin-4 and granulocyte-macrophage colony stimulating factor. The immature dendritic cells were also exposed to T9 cells that were killed by staurosporine-induced apoptosis, prolonged BK channel activation by phloretin, or heat shocked for 43°C for 5 minutes followed by an incubation for 18 hours in 0.9× PBS. Figure 12 shows that the dendritic cells displayed more maturation markers, MHC class II and CD86 antigens, when the cells were treated with BK channel activated/killed or with heat shocked T9 cells. In contrast, apoptotic T9 cells failed to stimulate dendritic cell maturation for MHC class II, but actually decreased CD86 expression. These data indicates that BK channel activated/killed T9 cells produce sufficient danger signals or other paraptosis-induced stimuli that cause the maturation of dendritic cells.

### T9 cells killed by prolonged exposure to BK channel activation stimulate T9 specific tumor immunity

**Figure 13:**
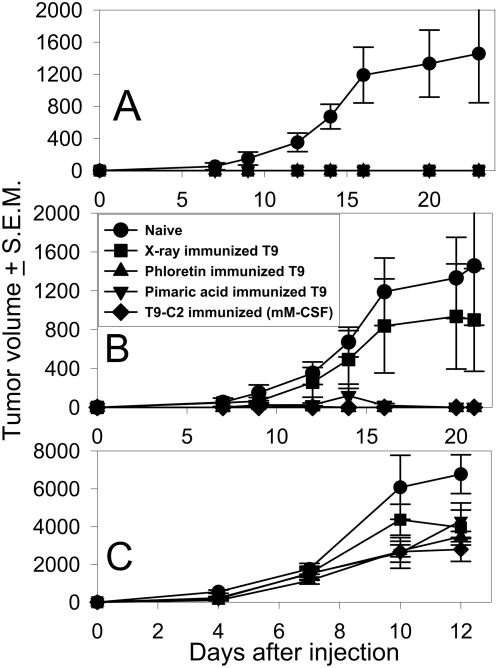
T9 cells killed by prolonged BK channel activation induce T9 specific immunity. Panel A: 10^6^ T9 cells were treated with 1 mM phloretin, or 0.01 mM pimaric acid for 12 hours, or 10G irradiated cells were injected subcutaneously into F344 rats. Tumor growth was then monitored. All rats in each group, either showed tumor growth (N = 8) or no tumor growth (N = 8). Panel B: The same immunized rats were rechallenged subcutaneously with 10^6^ T9 cells 25 days after the initial vaccination. The animals immunized by the phloretin-killed, pimaric acid-killed T9 cells or T9-C2 cell immunized rats were all statistically significantly different (P<0.05) from either the naïve control rats or the x-ray-killed immunized T9 rats. Twenty-three days later, the same rats were challenged with 10^5^ MADB106 breast cancer cells (Panel C). Each point represents 8 animals/group, except for the T9-C2 immunized rats (n = 4).

Because the prior experiments suggested that numerous “danger signals” were being expressed, an experiment was designed to test the hypothesis that T9 cells killed by prolonged BK channel activation could stimulate the immune system. *In vitro*, T9 cells were killed by prolonged BK channel activation (18 hours) using either phloretin or pimaric acid. Apoptotic, x-irradiated T9 cells were used as a negative control. T9-C2 cells were used as the living vaccine. As expected, none of the T9 cells killed by BK channel activation or living T9-C2 cells grew in subcutaneous sites (Figure 13, Panel A). The untreated T9 cells formed tumors. Three and a half weeks later the immunized rats were rechallenged (Panel B) with the injection of unmodified T9 cells. The rats that were immunized by exposure to T9 cells killed by BK channel activation (pimaric acid or phloretin), or by exposure to living T9-C2 cells demonstrated immunity. Non-immunized rats or rats immunized with x-irradiated T9 cells (n = 8) demonstrated a lack of immunity by the presence of growing T9 tumors. The immune results between the paraptotic T9 (phloretin or pimaric acid) vaccinated rats and the naïve or x-irradiated T9 vaccinated rats were significantly different (P<0.05). After this experiment was completed, all the rats that successfully rejected the T9 tumors were subsequently challenged with MADB106 breast cancer cells. Every rat that was injected with these unrelated syngeneic breast cancer cells formed subcutaneous tumors (Figure 13C). Thus, rats that were specifically immunized by BK channel activated/killed T9 cells produced immunity to T9 glioma cells, while the use of apoptotic T9 cells failed to generate sufficient immunity to reject a rechallenge with living T9 cells. This work demonstrates that induced paraptotic T9 cells killed by prolonged BK channel activation can reproduce the specific immunity seen when living mM-CSF transduced T9 cells are used as the initial immunogen.

## Discussion

We have hypothesized that the presence of the membrane form of M-CSF allows prolonged physical conjugation between transduced tumor cells and myeloid cells producing cytotoxicity that naturally facilitates anti-tumor immunity [25]. Supporting the development of this hypothesis are the following data from our previous works. A number of different tumor cells (rat T9 glioma cells [12]–[14], human U251 glioma cells [15], [20], rat MADB106 breast cancer cells [weak immunogenic, 26] and mouse Hepa1-6 hepatocellular carcinoma cells [non–immunogenic, 17]) that express the novel membrane form of M-CSF lack the ability to form either subcutaneous or intracranial tumors in rodents. Tumor cells transduced with the better known form of M-CSF, the secreted form of M-CSF (sM-CSF), were not killed by monocytes/macrophages *in vitro* [12] and were noted to form tumors in immunocompetent animals [16].

In all of the animal tumor models, it was observed that the animals were immunized to the unmodified parental tumor cells after being exposed to living mM-CSF transduced cells. After subcutaneous injection, membrane M-CSF transduced gliomas began dying within 4 hours of the development of the swelling and vacuolization process inherent in paraptosis [14], [15]. The molecular mechanism by which this cytotoxicity or immunity was mediated was never understood.

In the current study a putative molecular mechanism was investigated that facilitates an explanation of 1) how macrophages produce death in cloned rat mM-CSF expressing T9-C2 glioma cells; and 2) how tumor immunity is subsequently stimulated. In this study it was demonstrated that after 75 minutes of exposure, macrophages specifically responded to mM-CSF expressing tumor cells (Figure 1B) by releasing reactive oxygen species. The use of a broad caspase inhibitor, zVAD-fmk, failed to prevent the macrophage-mediated cytotoxicity of the T9-C2 cells (Figure 1C), suggesting that this death proceeds through a non-apoptotic pathway. Two monocyte-derived reactive oxidants (H_2_O_2_ and hypochlorite ions) proved to be effective cytolytic agents for the T9 cells. ROS scavengers also prevented the macrophages from causing the death of the mM-CSF target cells (Figure 1D).

Others have postulated that oxygen induces NADPH450 reductase and hemoxygenase-2 to make the secondary messenger, CO [24]. CO opens BK channels. The use of CO saturated media (Figure 4C) confirmed that cell swelling occurred within 15 minutes. The hypothesis was tested that within mM-CSF expressing tumor target cells prolonged activation of the BK channels by the respiratory burst of the macrophages mimics many of the actions of tumoricidal macrophages responding to mM-CSF expressing T9 tumor cells. This proposed cytolytic ROS mechanism explains how mM-CSF transduced tumor cells are specifically killed by rat macrophages and how this mechanism initiates anti-tumor immune responses by releasing “danger signals” similar to those derived from paraptotic cells [14], [15].

Within 60 minutes of treatment with two BK channel activators, phloretin and pimaric acid, the T9 glioma cells began swelling (Figure 3) and forming vacuoles (Figure S1). BK channels were detected on the cell membrane by immunofluorescence staining and confocal microscopy imaging (Figure 2E). Applied patch-clamping techniques proved these membrane BK channels to be functional (Figure 2A–D). With the use of co-localization immunofluorescence techniques, BK channels were observed in the ER and the mitochondria and these organelles were noted to swell in response to BK channel activators (Figure 3). Thus, it has been demonstrated for the first time that BK channels are functional in the ER. The mitochondria and the ER are affected in paraptosis and our findings provide a rationale for why these organelles are specifically targeted in this cellular death pathway. The demonstration of BK channels in the ER probably represents the normal synthesis and transport of BK channels towards the plasma membrane. The possibility also exists that targeted disruption of ER function leads to cellular death by disruption of protein synthesis.

When rat T9 glioma cells are compared with human U251 glioma cells, some notable differences are found. Even though both cells possess BK channels in the same locations [27], the U251 cells express more BK channels than T9 cells. This may explain why the U251 cells became swollen and vacuolated within 10–15 minutes, whereas, it took longer (60 minutes) to display the same cellular pathology in the T9 cells. The U251 cells took only 8–12 hours to die as a result of application of the phloretin or pimaric acid, while with the T9 cells required 17 hours (Figure 6A). These data suggests that there is a dose dependent relationship of these BK ion channels with down-stream effects. The T9 cells possess fewer BK channels, so a longer time is required to achieve the morphological changes observed with the U251 cells.

**Figure 14:**
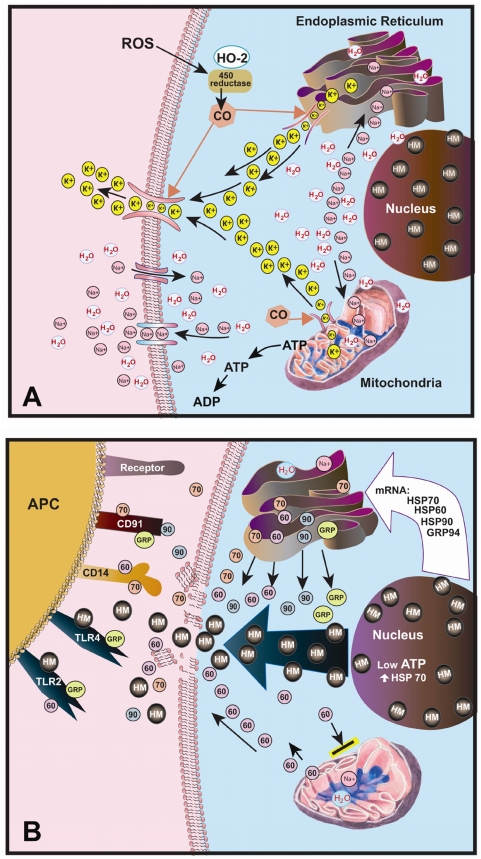
Proposed mechanism by which paraptosis and immunity are induced by prolonged BK channel activation. Panel A shows the mechanism by which paraptosis is induced by ionic disregulation via prolonged BK channel activation. Panel B shows the results of the ionic disregulation that induce immune responses by stimulating antigen presenting cells (APC). The precise topology of the binding sites for the HSPs and HMGB1 to the receptors found on the APC should not be inferred from this diagram.

The presented model of paraptosis induction contains six steps in Figure 14, Panel A. Before macrophages come into contact with the mM-CSF tumor cells, normal homeostasis is present. Intracellular ATP, K^+^ and Na^+^ are at normal baseline physiological levels; i.e., intracellular concentrations that are high in ATP and K^+^ but low in Na^+^. When macrophages encounter the mM-CSF cell via the M-CSF receptor, reactive oxygen species, such as H_2_O_2_ and HOCl, are produced (Step 1). The presence of ROS allows hemoxygenase and P450 reductase to enzymatically produce CO (Step 2) as a secondary signal. CO mediates the opening of BK channels (Step 3) in the cell membrane, as well as in the ER and the mitochondria thereby allowing pooled stores of K^+^ to be expelled. As the cell releases K^+^ (Step 4), Na^+^ cations enter via another inward Na^+^ specific channel, preserving the electroneutrality of the cell. When Na^+^ enters the cell (Step 5), water follows, producing the observed cellular swelling. Because of the extra Na^+^ and water, vacuolization occurs during ER and mitochondria swelling (Step 5). Cellular homeostatic mechanisms are activated in an effort to expel the excess intracellular Na^+^ through the ATP dependent Na^+^/H^+^ antiporter [28] or the Na^+^/K^+^ ATPase pump (Step 6). Inhibition of the Na^+^/H^+^ antiporter induced paraptosis in cerebellar neurons [29]. When the BK channels are fully engaged in response to the macrophage mediators, the cell remains swollen. The cell expends more ATP in an effort to expel the unwanted Na^+^ ions. Since the mitochondria are targeted in paraptosis, functional disruption of this organelle reduces the cell's ability to generate sufficient levels of ATP to maintain ionic homeostasis. Several groups have reported that the loss of ATP within cells results in the development of a necrosis forming pathway [30]–[33]. Attacking the generation of ATP, without directly inducing apoptosis, eventually prevents the cell from maintaining the necessary intracellular volume. Catastrophic failure in ATP production leads to decreases in function that expel Na^+^ with subsequent osmotic rupture of the cell. The Cr^51^ release data indicated that actual membrane rupture occurred at 17 hours (Figure 7A). Therefore simple cellular swelling and vacuolization are insufficient to induce immediate cell death. Further downstream events are required. This is consistent with the slow depletion of intracellular ATP described in our predicted model. Figure 7 demonstrates that intracellular ATP levels did indeed drop as the cells died. When the luciferase transduced T9-C2 cells were used, intracellular ATP levels declined in a time-dependent manner, as macrophages began attacking the target cells. This time correlated very well with the actual time that Cr^51^ lysis occurred at 17 hours.

Several research groups have reported an association of depleted intracellular ATP levels with increased heat shock protein (HSP) transcription and expression [34], [35]. Hence the extended time required for ATP depletion by this BK mediated paraptosis permitted the heat shock response to occur. The data in this study proved that HSP expression occurs in response to either phloretin, pimaric acid, or hydrogen peroxide (Figure 8). Immunohistology revealed that the paraptotic mM-CSF transduced glioma cells demonstrated an increased expression of three different HSPs (Hsp60, 70 and GRP94/gp96) family members after monocytes attacked these target cells in vivo [14], [15]. The works of other researchers confirmed that necrotic cells also over-express several HSPs [4], [8], [36]. HSPs have been described as possible “danger signals” [4] for necrotic cell death. Released HSPs may then stimulate the immune system to become activated against the host associated antigens presented by paraptotic cells. Hsp70, Hsp90 and GRP94/gp96 have increased the immunogenicity of tumors by increasing the synthesis of tumor antigens recognized by the T cells [8], [37]. Once the tumor cell releases these HSPs, the host's antigen presenting dendritic cells, can take up these antigens via the CD14, CD91, TLR2 and 4 receptors [8], [38]. Hsp70 and GRP94/gp96 enhanced dendritic cell maturation [39], [40]; Hsp70 acted as a cytokine, stimulating tumor necrosis factor, interleukin-1 and interleukin-6 production from CD14+ monocytes and immature dendritic cells [41]. Tumor antigens maybe released when the tumor cell lyses, or during the normal degradation process when HSP assist in proteosomal degradation of the peptide fragments. Gene therapy of murine RM-9 prostate cancer cells with the nitroreductase gene (NTR), followed by administration of the pro-drug, Hsp25 and Hsp70 expression was induced and the targeted cells died via a necrosis-like pathway [42]. As a result of combined NTR and HSP70 adenoviral therapy, better CD4 and CD8 T cell immunity were stimulated.

Another danger signal that we saw becoming mobilized after BK channel activation was HMGB1, normally a nuclear/perinuclear protein (Figure 10). Upon stimulation with either phloretin or pimaric acid, HMGB1 was translocated from the perinuclear location to the membranes and was even detected on the exterior of the treated T9 cells (Figure 10, Panel E). HMGB1 translocates from the nucleus to the membrane only during necrosis and not during apoptosis [3].

Since many danger signals were capable of being made, T9 cells were killed by prolonged BK channel activation and then used as a prophylactic vaccine in rats (Figure 11). All rats that were vaccinated with the BK channel activated/killed T9 cells or T9-C2 cells produced specific immunity towards the T9 glioma cells (Figure 11B) but not towards an unrelated MADB106 breast cancer cell line (Figure 11C).

Figure 14B exemplifies the mechanism by which paraptotic cells eventually release the “danger signals” as the cell swells and eventually ruptures. Overall these released “danger signals” can stimulate the APC that are needed for tumor immunity, which is subsequently produced after mM-CSF cells are killed by the macrophages. Thus, the paraptotic cells can release several factors that elicit an “immunostimulatory storm”, strongly activating the APC and producing anti-tumor immunity.

The work reported here demonstrates for the first time, how tumor cells killed by BK channel-induced ionic disregulation might be used as a non-genetically modified glioma cell vaccine. Our work explains the molecular mechanisms by which the mM-CSF transduced cells elicited systemic immunity via multiple danger signal production. Previously all the positive therapeutic results produced by using mM-CSF transduced cells were generated using live tumor cells [14]–[18], [25], [26]. Previously, when mitomycin-c treated, x-irradiated or frozen T9-C2 cells were used as a vaccine; little systemic immunity was demonstrable when compared to the living T9-C2 vaccine [14]. Translating a living mM-CSF based vaccine into a viable treatment in a human clinical setting would be problematic, for the obvious reason of injecting living tumor cells into humans. With the discovery of this mechanism of mM-CSF tumor cell death, clinical trials can be designed in which tumor cells are first killed through activation of paraptosis-inducing pathways; i.e. BK channel activation or via release of reactive oxygen species. The advantage of this proposed approach is that one could easily kill the tumor cells by prolonged BK activation in vitro and use the treated cells as a functional killed vaccine, without any further genetic manipulation. Hence, once freshly isolated tumor cells possessing BK channels are produced, these cells could quickly be used as an autologous vaccine. Such a possibility marks this finding as having immediate and significant clinical potential for successful treatment and vaccine development for cancer.

## Materials and Methods

### Cell lines and cell culture

The rat T9, MADB106 and mM-CSF transduced T9 glioma cells have been previously described [14]–[16]. All culture supplies were screened and selected on the basis of being under the limits of detection by limulus amebeocyte lysate assay.

### Chemicals

Phloretin was purchased from Sigma Chemical Corp (St. Louis, MO). Pimaric acid and recombinant iberiotoxin were obtained from Alomone Labs (Jerusalem, Israel). zVAD was graciously provided to us by Dr. Kirston Koths, Chiron Corp. (Emeryville, CA).

### Animals

Fisher F344 rats were obtained from Charles River (Wilmington, MA). The animals were housed in our AAALAC accredited facility. Animal experimentation procedures were carried out according to the Animal Studies Committee approved protocols.

### Electron microscopy

Five thousand T9 cells were plated onto sterile gelatin coated coverslips within 24-well plates. After an overnight incubation, the cells were treated with either phloretin, pimaric acid or with regular tissue culture media for 1 hour at 37°C. The cells were fixed at room temperature in 1% glutaraldehyde in Sorensen's phosphate buffer pH 7.3 for 10 minutes and brought to 4°C during fixation. The samples were rinsed by 3-one minute washes in cold Sorensen's buffer, followed by 15 minute post-fixation in cold 1% OsO4, and rinsed again. Following a 2 minute dehydration with a graded series of cold ethanol (30%, 50%, 75%, 95% and 100%), the samples were brought to room temperature (over a 5 minute time) treated with four 15 second changes of 100% ethanol, two 15 second changes of propylene oxide and then transferred to a 50/50 mix of propylene oxide/araldite 502 for 15 minutes. Coverslips were then dipped into pure plastic until it appeared that most of the propylene oxide was gone and then the coverslips were laid cell side down onto plastic molds containing araldite. After 36 hours of incubation at 60°C the coverslips were detached from the polymerized specimens by immersion into liquid nitrogen. Areas of interest were super glued onto plastic stud blocks and then trimmed and cut in planar sections. The sections were stained with uranyl acetate and lead citrate and then observed using a JEOL-1200EX II transmission electron microscope (Peabody, MA). Digital micrographs were taken with a Hammamatsu C4742-95 camera.

### Isolation of macrophages

Rats were injected with 5 mls of sterile endotoxin-free 3% thioglycollate solution 2 days prior to experiment induction. The treated rats were euthanized and the peritoneum were washed using sterile endotoxin free saline irrigational solution.

### Preparation of dendritic cells

Femurs were removed from euthanized rats and flushed with endotoxin-free PBS. Bone marrow cells were cultivated in vitro for 3 days in complete RPMI-1640 (Invitrogen, Carlsbad, CA) supplemented with 1,000 U/ml each of recombinant rat interleukin-4 (IL-4) (Biosource/Invitrogen, Carlsbad) and recombinant rat granulocyte-macrophage colony stimulating factor (GM-CSF) (Biosource/Invitrogen, Carlsbad). The dendritic cell cultures were exposed for 1 day to the various preparations of killed T9 cells. Apoptotic cells were killed by an 18 hour exposure to 10 µM staurosporine, while paraptosis induction used 1 mM phloretin. In addition, a positive control was generated by exposure of the dendritic cells to 500 ng/ml lipopolysaccharide. The dendritic cells were detached by incubating the cells in versene/phosphate buffered saline (PBS) for 30–60 minutes at 4°C. The cells were dislodged using a cell scraper. This procedure resulted in >95% viability of the cells.

Dendritic cells (10^6^ cells) were then stained for the presence of maturation markers (CD86, and MHC class II) for 1 hour on ice. All direct labeled antibodies were purchased from eBiosciences (San Diego, CA). Ten thousand cells were analyzed on the Facs Calibur (Bectin-Dickson) flow cytometer. The mean channel number of the dendritic cells was then compared.

### Cytotoxicity

Cytotoxicity studies were performed according to previously reported methods using a radio-isotope based assay [16]–[20]. Ten thousand target cells were incubated in 200 µl of media in a humidified, 5% CO_2_ incubator for 24 hours. Cytotoxicity data from quadruplicate cultures of each macrophage were collected.
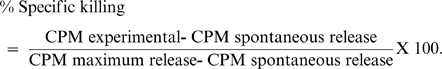
The tumor cell killing is presented as mean values±standard deviations.

### Reactive Oxygen Species (ROS) Detection

Monocytes were pre-labeled with 1 µM H_2_DCFDA (Molecular Probes, Eugene, Or) for 20 minutes at 37°C using the manufacturer's directions. The cells were washed three times then added at a 1∶1 monocyte∶tumor concentration in sextuple replicates. The samples were then analyzed using a Novostar Luminometer/Fluorometer (BMG Labtech, Offenburg, Germany).

### Electrophysiology

Typical patch-clamp techniques were used to record single-channel and macroscopic membrane currents as described earlier [27], [42].

### Immunofluorescence/Confocal Microscopy

Adherent T9 glioma cells on sterile cover glass were cultured in RPMI media without serum. The cells were fixed with 2% paraformaldehyde, permeated in 0.2% Triton-X and probed with either rabbit anti-BK [also called: K_Ca1.1_ (BK_Ca_)] antibody (Alomone Labs, Jerusalem, Israel) or anti-HMGB1 (StressGen, Victoria, Canada). Mitochondria were stained with MitoTracker Deep red (Molecular BioProbes/Invitrogen, Eugene, Oregon). The endoplasmic reticulum was initially stained with goat anti-GRP78 antibody (Santa Cruz Biotech, Santa Cruz, CA). Later experiments used ER-tracker (Molecular BioProbes/Invitrogen, Eugene, Oregon). The cover glass was incubated for 2 hours with the primary antibody in a humidified chamber at 4°C. The slides were then washed three times in PBS. After treatment with secondary fluorescein anti-rabbit antibody or Texas Red anti-goat antibody (Vector Labs, Burlingame CA) the slides were incubated for sixty minutes. Finally, the cells were washed three times in PBS and mounted with ProLong Gold antifade reagent (Invitrogen, Carlsbad, CA). Under certain circumstances cells were counterstained with nuclei dyes. Molecular BioProbes/Invitrogen nuclear counterstaining kits with Sytox Green or Hoechst 33342 blue dye were used according to the manufacturers directions. Samples were imaged using a Nikon two laser (HeNe and Argon) PCM 2000 Confocal System on an Eclipse E800 Microscope. The two different fluorescent dyes in the labeled sample were simultaneously acquired through a single illumination and detection pinhole using Compix Simple PCI software as previously reported [21]. This provided exact pixel for pixel registration in both time and space for each dye in each channel. As a result, it can be inferred that the red emitting and green emitting probes are colorized when yellow areas are present in the images shown.

### siRNA and Real-Time Polymerase Chain Reaction Analysis

Invitrogen Stealth siRNA ™ dsRNA technology was used. Two Stealth siRNA primers (5′-UUU AAG UAU ACA GAC ACA AAC ACG G and 5′-CCG UGU UUG UGU CUG UAU ACU UAA A) were designed specifically for rat BK channels. The control SiRNA (1027280) was purchased from Qiagen. The primers (80 pmol) were complexed with lipofectamine and incubated with T9 cells in six well plates (40,000 cells/well) under serum-free and antibiotic-free culture conditions for 1 hour. The cells were then washed with PBS and cultured in complete RPMI media. The kinetics of mRNA inhibition was assessed after the transfection. On each day after transfection, the cells were lysed and total RNA collected. The RNA was treated with DNAse and then the DNAse was removed by a phenol extraction. The RNA was converted into cDNA by utilizing reverse transcriptase, followed by real-time PCR techniques using 18S and BKα channel specific real-time primers (forward: 5′ GAT TGA GGA AGA CAC ATG G and reverse: 5′ CAG CTC ACA AAC AGT AGG). Real-time PCR reactions were performed on an iCycler iQ detection system using a Brilliant SYBR Green kit (Stratagene, San Diego, CA).

### ATP determination

In sextuplicates, twenty thousand T9 cells were cultured in each well of a 96 well plate under the various experimental conditions described in the figure legends. After 1, 16 or 24 hours of incubation the cells were collected, lysed and then the amount of ATP present was measured. The measurement of ATP levels was carried out using the Molecular Probes' ATP Determination Kit according to the manufacturer's directions. Bioluminescence was measured using a Novastar Luminometer/Fluorometer.

### Luciferase Assay for determining intracellular ATP levels

The pDON-luciferase producing retrovirus was obtained from Dr. W.F. Anderson (USC School of Medicine, Los Angles, CA)[43]. T9 and T9-C2 cells were transduced with this retrovirus. These luciferase transduced cells were designated as T9/luc+ of T9-C2/luc+, respectively. Afterwards, these cells were cloned by limiting dilution. Clones were screened by taking 10^4^ cells/well in a 96 well plate format and adding in 100 µl of luciferin (Invitrogen). The luminescence was measured in the Novostar Fluorometer (BMG Labtech, Offenburg, Germany). T9/luc+ and T9-C2/luc+ clones that displayed luminescence units (>10^6^ luminescence units) were selected for the macrophage mediated cytotoxicity. The mM-CSF expression as detected by flow cytometry of T9-C2/luc clones was not different from that of the parental T9-C2 cells.

Freshly isolated thioglycollate elicited macrophages were collected and allowed to adhere onto the 96 well plates (10^5^ cells/well) for 2 hours at 37°C. The T9/luc+ and T9-C2/luc+ (10^4^ cells) were added to the macrophage containing wells or to empty wells which served as controls. All assays were done in quadruplicate cultures. Immediately after the cells were combined, a set of cells was added with luciferin to obtain baseline values using the Novostar Fluorometer. Then after 1, 2, 6, 12 and 18 hours, the process was repeated and the kinetics of luminescence was measured. Since the T9/luc clones displayed a higher baseline luminescence value (120,000 units/10^5^ cells) than T9-C2/luc clones (100,000 units/10^5^ cells), we normalized the value values to account for the difference in Figure 8.

### Intracellular flow cytometry

Exponentially growing T9 glioma cells were prepared with the reagents and protocols of Santa Cruz Biotechnology (Santa Cruz, CA). The cells were either treated with the BK channel activators or heat shocked at 43°C for 5 minutes and incubated at 37°C for 6 hours. The cells were trypsinized and washed. The fixed cells were washed twice in ice-cold PBS. The cells were permeabilized for 15 minutes on ice. The cells were washed twice. The resuspended cells were then divided into 10^6^ cell aliquots and then incubated with the primary antibody for 1 hour. The anti-HSP antibodies (Hsp60, 70, and Grp94/gp96 were obtained from (Stressgen, Victoria, Canada), while the anti-Hsp90 antibody was purchased from Santa Cruz Biotechnology (Santa Cruz, CA). The cells were washed twice and the secondary antibody-conjugated with fluorescein isothiocyanate (FITC) (Vector Labs, Burlingame, CA) was incubated on ice for another hour. After washing the cells twice, the cells were analyzed with a Bectin-Dickson FacsCalibur flow cytometer. The staining profiles were recorded at the same FL1 PMT voltage.

### Tumor growth

Manually restrained rats were injected subcutaneously with 10^6^ tumor cells in a volume of 100 µl. The resultant tumors were measured with metric calipers three times a week. Data recorded included the length, width and height of the tumors. Tumor volumes were calculated using the following equation: volume = length×width×height×π/6. The data were expressed as the mean tumor volume.

### Statistics

A Student's t test was used to analyze all *in vitro* data. The animal data was analyzed by using a Fisher's exact test. A P value of <0.05 was considered significantly different from control values.

## Supporting Information

Figure S1 BK channel activators produced vacuolization of mitochondria and ER within the T9 cells within 1 hour. Panels A, D and G shows adherent untreated, control T9 cells. T9 cells incubated for 1 hour in phloretin (1 mM) (Panels B, E and H). Panels C, F and I illustrate T9 glioma cells treated for 1 hour with 0.01 mM pimaric acid. T9 cells were pre-labeled with either Mito-Tracker (Panels D, E and F) or with ER-Tracker (Panels G, H and I). Panel J shows the increases in pixel number of the cells derived from 10 different cells under each condition. Asterisks indicate significant differences (P<0.05) from their respective controls.
(9.14 MB TIF)

Figure S2 Mitochondria within the T9-C2 cells swell in response to the effects of the macrophages. T9 and T9-C2 cells were pre-labeled with Mito-Tracker and then incubated with rat macrophages for 4 hours at 37°C. Panel A shows the macrophages (indicated by arrows) attaching themselves to T9 cells. The T9 cells' mitochondria appear normal. Panel B shows the T9-C2 cell also conjugated to 2 macrophages. In contrast, the T9-C2 mitochondria appear swollen. Magnification 200×.
(7.40 MB TIF)

Figure S3 Mouse peritoneal macrophages are prevented from killing the mM-CSF expressing T9-C2 cells by a BK channel inhibitor, iberiotoxin. Peritoneal macrophages elicited by thioglycollate after 2 days were incubated at a 10∶1 macrophage∶tumor ratio (quadruplicate cultures) for 24 hours. Recombinant iberiotoxin (0.05 µM) was added to 1 set of the macrophage: tumor cells at time 0.
(8.29 MB TIF)
