# Supplementary figures and images for: Molecular Mechanisms of Paraptosis Induction: Implications for a Non-Genetically Modified Tumor Vaccine
Source: PLoS One. 2009 Feb 27;4(2):e4631. doi: 10.1371/journal.pone.0004631 (PMC2645013; doi:10.1371/journal.pone.0004631)

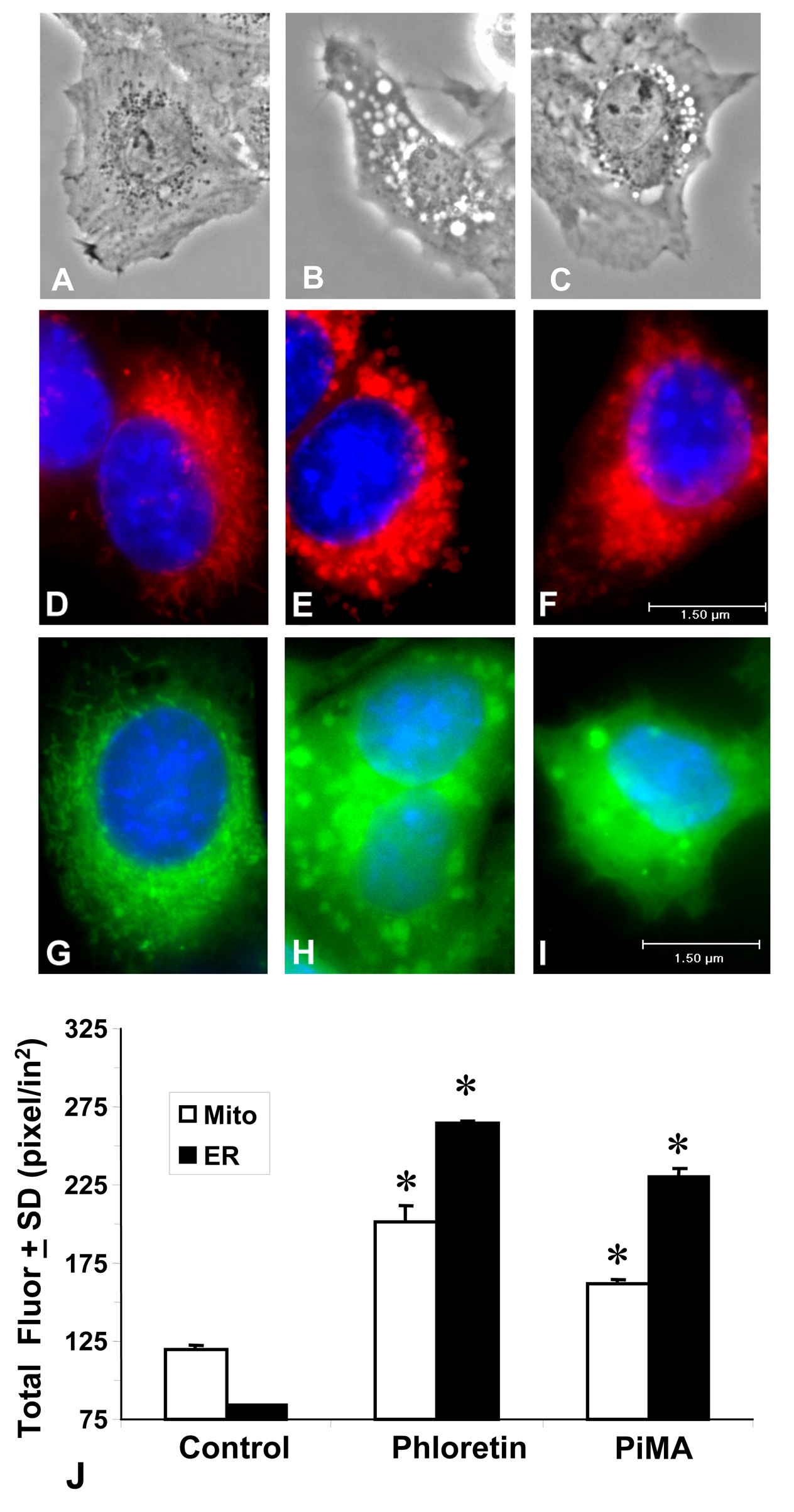

Supplement: Figure S1 — BK channel activators produced vacuolization of mitochondria and ER within the T9 cells within 1 hour. Panels A, D and G shows adherent untreated, control T9 cells. T9 cells incubated for 1 hour in phloretin (1 mM) (Panels B, E and H). Panels C, F and I illustrate T9 glioma cells treated for 1 hour with 0.01 mM pimaric acid. T9 cells were pre-labeled with either Mito-Tracker (Panels D, E and F) or with ER-Tracker (Panels G, H and I). Panel J shows the increases in pixel number of the cells derived from 10 different cells under each condition. Asterisks indicate significant differences (P<0.05) from their respective controls. (9.14 MB TIF) [file pone.0004631.s001.tif]

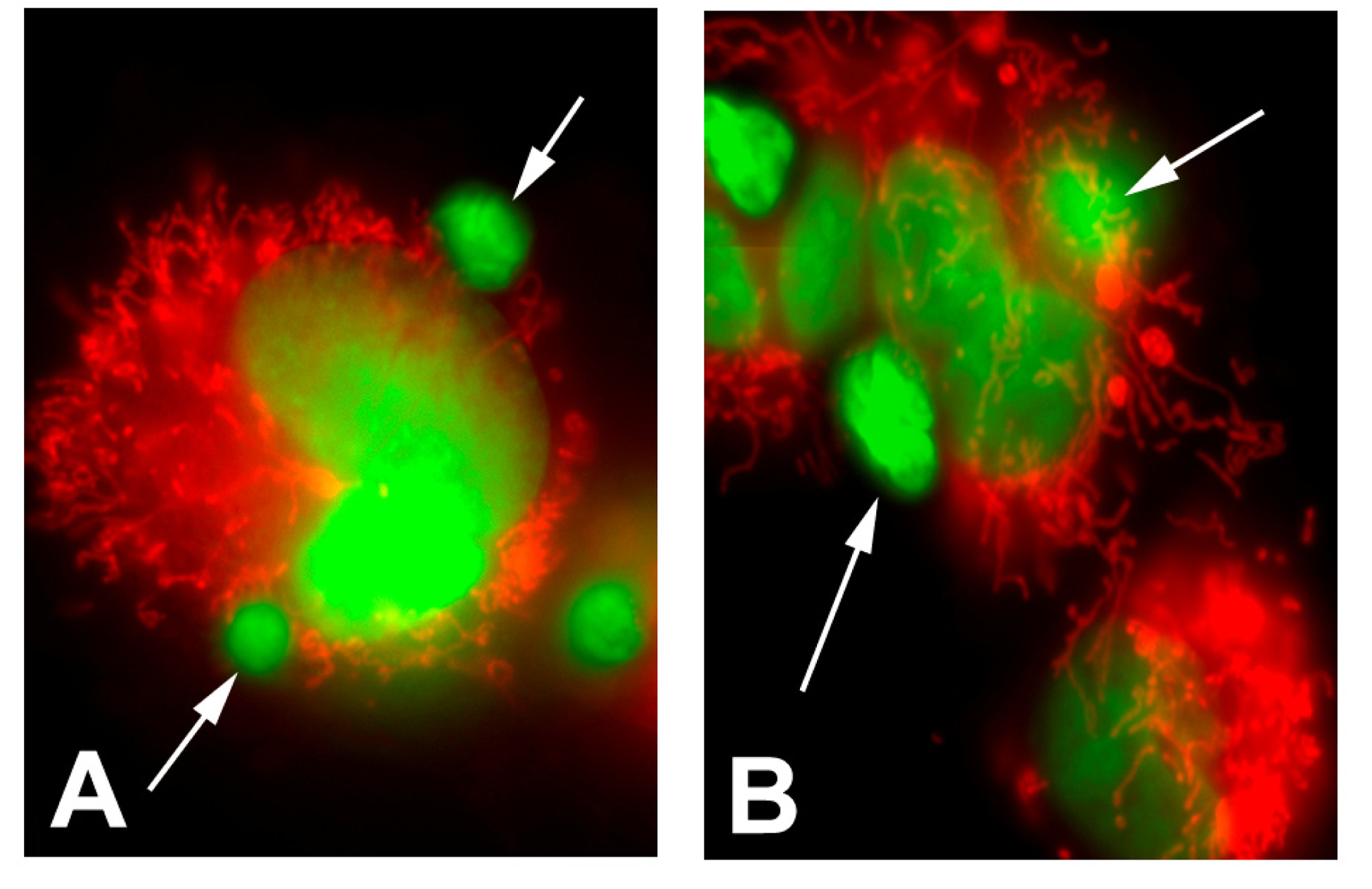

Supplement: Figure S2 — Mitochondria within the T9-C2 cells swell in response to the effects of the macrophages. T9 and T9-C2 cells were pre-labeled with Mito-Tracker and then incubated with rat macrophages for 4 hours at 37°C. Panel A shows the macrophages (indicated by arrows) attaching themselves to T9 cells. The T9 cells' mitochondria appear normal. Panel B shows the T9-C2 cell also conjugated to 2 macrophages. In contrast, the T9-C2 mitochondria appear swollen. Magnification 200×. (7.40 MB TIF) [file pone.0004631.s002.tif]

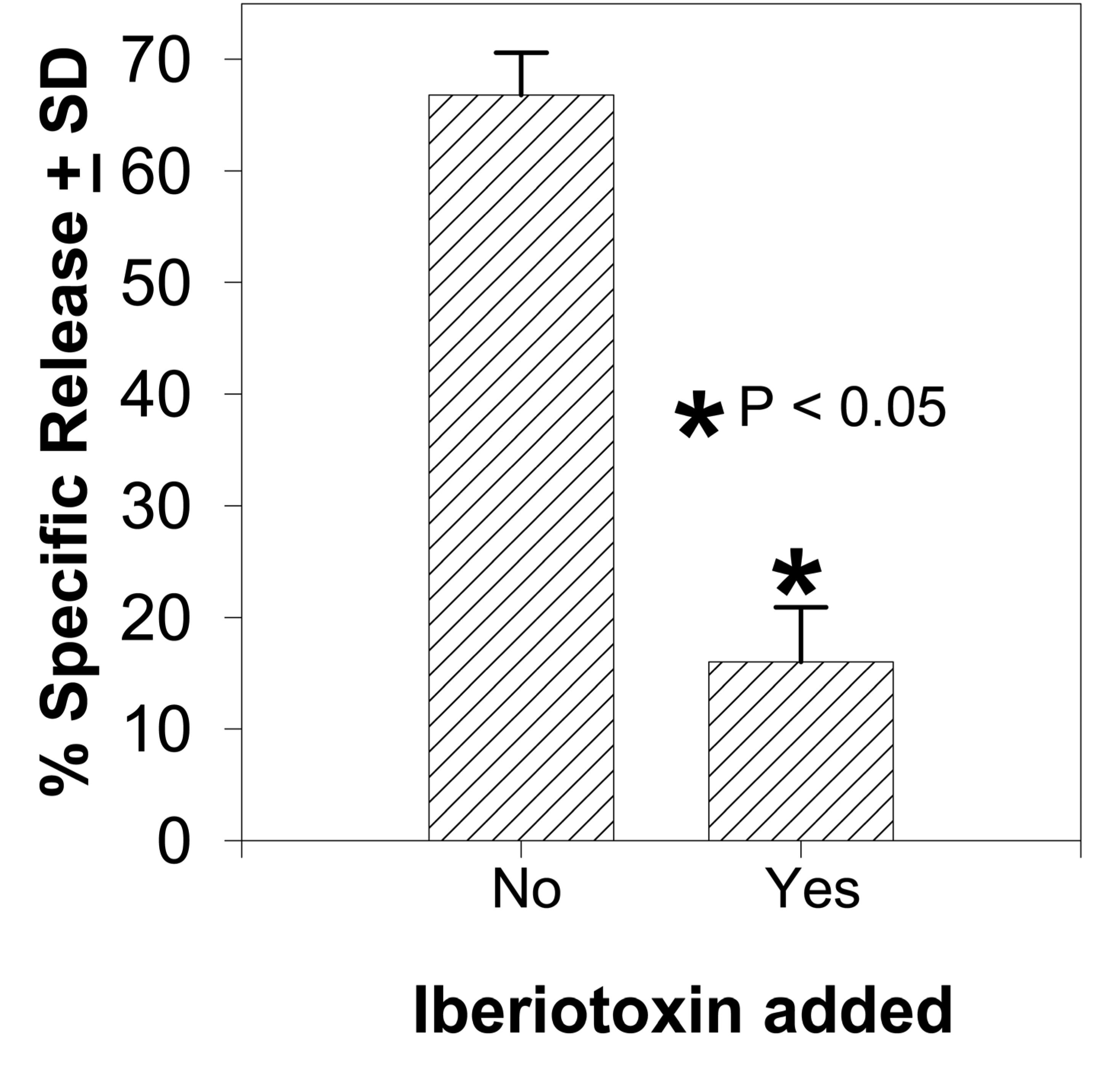

Supplement: Figure S3 — Mouse peritoneal macrophages are prevented from killing the mM-CSF expressing T9-C2 cells by a BK channel inhibitor, iberiotoxin. Peritoneal macrophages elicited by thioglycollate after 2 days were incubated at a 10∶1 macrophage∶tumor ratio (quadruplicate cultures) for 24 hours. Recombinant iberiotoxin (0.05 µM) was added to 1 set of the macrophage: tumor cells at time 0. (8.29 MB TIF) [file pone.0004631.s003.tif]
